# Effective strategies to reduce commercial tobacco use in Indigenous communities globally: A systematic review

**DOI:** 10.1186/s12889-015-2645-x

**Published:** 2016-01-11

**Authors:** Alexa Minichiello, Ayla R. F. Lefkowitz, Michelle Firestone, Janet K. Smylie, Robert Schwartz

**Affiliations:** 1 Ontario Tobacco Research Unit, Dalla Lana School of Public Health, University of Toronto, Toronto, Canada; 2 Well Living House Action Research Centre for Indigenous Infant, Child and Family Health and Wellbeing,, St. Michael’s Hospital, Toronto, Canada; 3 Centre for Research on Inner City Health, St. Michael’s Hospital, Toronto, Canada; 4 Dalla Lana School of Public Health, University of Toronto, Toronto, Canada

**Keywords:** Indigenous, International, Commercial tobacco, Interventions, Systematic review

## Abstract

**Background:**

All over the world, Indigenous populations have remarkably high rates of commercial tobacco use compared to non-Indigenous groups. The high rates of commercial tobacco use in Indigenous populations have led to a variety of health issues and lower life expectancy than the general population. The objectives of this systematic review were to investigate changes in the initiation, consumption and quit rates of commercial tobacco use as well as changes in knowledge, prevalence, community interest, and smoke-free environments in Indigenous populations. We also aimed to understand which interventions had broad reach, what the common elements that supported positive change were and how Aboriginal self-determination was reflected in program implementation.

**Methods:**

We undertook a systematic review of peer-reviewed publications and grey literature selected from seven databases and 43 electronic sources. We included studies between 1994 and 2015 if they addressed an intervention (including provision of a health service or program, education or training programs) aimed to reduce the use of commercial tobacco use in Indigenous communities globally. Systematic cross-regional canvassing of informants in Canada and internationally with knowledge of Indigenous health and/or tobacco control provided further leads about commercial tobacco reduction interventions. We extracted data on program characteristics, study design and learnings including successes and challenges.

**Results:**

In the process of this review, we investigated 73 commercial tobacco control interventions in Indigenous communities globally. These interventions incorporated a myriad of activities to reduce, cease or protect Indigenous peoples from the harms of commercial tobacco use. Interventions were successful in producing positive changes in initiation, consumption and quit rates. Interventions also facilitated increases in the number of smoke-free environments, greater understandings of the harms of commercial tobacco use and a growing community interest in addressing the high rates of commercial tobacco use. Interventions were unable to produce any measured change in prevalence rates.

**Conclusions:**

The extent of this research in Indigenous communities globally suggests a growing prioritization and readiness to address the high rates of commercial tobacco use through the use of both comprehensive and tailored interventions. A comprehensive approach that uses multiple activities, the centring of Aboriginal leadership, long term community investments, and the provision of culturally appropriate health materials and activities appear to have an important influence in producing desired change.

**Electronic supplementary material:**

The online version of this article (doi:10.1186/s12889-015-2645-x) contains supplementary material, which is available to authorized users.

## Background

### The issue

All over the world, Indigenous^1^ populations have remarkably high rates of commercial tobacco use compared to non-Indigenous groups [1]. For example, the prevalence of commercial tobacco use in Australia, New Zealand and the United States (US) is 16, 15, and 18 % respectively, in contrast to the rates for Australian Aboriginal and Torres Strait Islander peoples (42 %), New Zealand Māori peoples (39 %), and American Indians and Alaska Natives (22 %) [2–4]. In Canada, the rate of commercial tobacco use in the general population is 18 % [5], while in the First Nations (off-reserve), First Nations (on-reserve), Inuit and Métis populations the rates are 35.8, 59.0, 59.8 and 33.0 % respectively [6, 7]. The high rates of commercial tobacco use in Indigenous populations have led to a variety of health issues and lower life expectancy than the general population [8]. These health disparities have focused the attention of many Indigenous communities, national and regional governments on efforts to reduce commercial tobacco use [9].

In the general population, comprehensive tobacco control strategies have demonstrated positive changes in reducing tobacco consumption. For example, Canada’s Federal Tobacco Strategy has used a variety of different interventions, such as educational campaigns, a quitline and regulating sales, and has seen a 6 % reduction in tobacco use nation-wide over 11 years [9]. While these interventions have positively affected the general population, Aboriginal peoples in Canada have not experienced the same reduction in commercial tobacco use [9]. These health disparities are further compounded by a history of colonialism and social disadvantage for Indigenous peoples across the world [10].

To better inform the development of future policies and programs, we conducted a systematic review of literature that examined which commercial tobacco prevention, cessation, and protection interventions have led to positive changes among Indigenous populations worldwide. In this review, commercial tobacco use is distinguished from the ceremonial use of tobacco which is considered a sacred medicine for many First Nations people in Canada since pre-colonization.

### Gap in the literature

There is scarce published literature reviewing interventions aimed at reducing commercial tobacco use in Indigenous communities globally. DiGiacomo et al. [11] and Carson et al.’s systematic reviews [1, 12] are the only international examinations of the effectiveness of commercial tobacco control interventions for Indigenous populations. DiGiacomo et al. limited their search to individual level interventions, which are interventions in which participants interact directly with health professionals, and focused solely on quit rates [11]. In both their 2013 and 2014 papers, Carson et al. focused their reviews on Aboriginal and Torres Strait Islander peoples in Australia and included only randomized or quasi-randomized controlled trials [12] and pre and post-studies and reports [1].

This systematic review adds to this literature in the following ways. First, it provides an international analysis of commercial tobacco control interventions for Indigenous people, without an explicit focus on Australian interventions, with 34 % of studies from the US and 19 % from Canada. Second, this review evaluates a variety of outcome measures not included in previous reviews, including change in community interest, prevalence, consumption, quit rates, initiation, knowledge and smoke-free environments. Third, the inclusion of both qualitative and quantitative literature allowed us to review the impact of a multitude of interventions, some of which were only evaluated using qualitative research methods. And fourth, this review has an emphasis on using Indigenous defined measures of change, recognizing the need to include Indigenous peoples and their self-defined priorities and needs in the planning, management, and evaluation of Indigenous health programs and services [13, 14].

### Project aims

Central to our analysis is the viewpoint that effective health services and programs in Indigenous communities are self-managed and appropriate to local contexts, knowledge systems and skills [15].

The particular objectives of the review were to:

- Assess change in the following outcomes: community interest, knowledge, rates of initiation, rates of consumption, quit rates, the presence of smoke-free environments and prevalence of commercial tobacco use in Indigenous communities.
- Understand which interventions demonstrated community level change. Community level change is measured through the use of population level measurements such as community censuses. It occurs when change has been measured and a difference has been observed within the population and not just in individuals.
- Uncover common elements or strategies that prove effective in producing desired changes in Indigenous communities noting specific contexts in which these elements/ strategies were or were not effective.
- Explore the ways that services and programs reflect Aboriginal self-determination, defined as “the incorporation of Indigenous beliefs, knowledge and skills at the centre rather than at the margins of Indigenous health policy, programming and service delivery” [15].

## Methods

This review is one component of a larger community-based research project working to create knowledge to help create effective commercial tobacco reduction interventions in Aboriginal communities in Ontario, Canada. We searched peer reviewed and grey literature from databases and electronic sources. Systematic cross-regional canvassing of informants in Canada and internationally with knowledge of Indigenous health and/or tobacco control provided further leads about commercial tobacco reduction interventions. A broad range of commercial tobacco control interventions from six different countries were retrieved.

### Search strategy

Our search terms were divided into three categories to represent the themes we were looking for: Indigenous search terms, tobacco use search terms and intervention search terms. Several combinations of search terms were used and altered depending on the database and the items found. See Table 1 for a full list of search terms.

**Table 1:** List of search terms

*Indigenous Search Terms:*	“Aborigin*” or “Indigenous” or “Native” or “Eskimo*” or “Inuit*” or “Inuk*” or “Metis” or “First Nations” or “Native Canadian*” or “Native American” or “Maori*” or “Pacific Islander” or “American Indian*” or “Native Alaska*” or “Alaska Native*” or “Native Hawaiian*” or “Torres Strait Islander*” or “Yupik” or “Aleut”
*Tobacco Use Search Terms:*	“tobacco” or “smoke” or “smoking” or “cigarettes” or “tobacco use” or “cigar”
*Intervention Search Terms:*	“smoking prevention” or “tobacco control” or “smoking cessation” or “smoking reduction” or “intervention” or “program” or “initiative” or “program evaluation” or “tobacco reduction” or “tax” or “smoking ban” or “smoking restriction” or “tobacco reduction strateg*” or “tobacco control strateg*” or “quit smoking”

We searched seven databases of peer reviewed literature, including Embase, Ovid MEDLINE, PsychINFO, CINAHL, Social Service Abstracts, Social Work Abstracts and Web of Science, as well as 43 electronic sources for grey literature. A full list of databases and electronic sources can be found in Table 2.

**Table 2:** List of Databases

*Peer-reviewed Sources:*	*Grey Literature Sources:*
Embase,	Australian Indigenous HealthInfoNet,
Ovid Medline,	Bibliography of Native North Americans,
PsychINFO,	First Nations Health Council,
CINAHL,	Circumpolar Health bibliographic Database,
Social Work Abstracts,	Native Health Database,
Web of Science,	I-Portal Indigenous Studies Portal,
ROVER,	BIOSIS Previews,
Scopus	American Indian Health,
	Arctic Health Publications Database,
	DARE – York University,
	CADTH,
	Canadian Women's Health Network,
	Centre for Excellence in Indigenous Tobacco Control,
	Centre for Indigenous Environmental Resources,
	Centre for Inuit Health and Changing Environments,
	Centres for American Indian and Alaskan Native Health,
	Inuit Tobacco Free Network,
	Journal of Aboriginal Health,
	Lowitja Institute,
	Menzies School of Health Research,
	Metis Health Research Database,
	NAHO,
	New Brunswick Anti Tobacco Coalition,
	NCCAH,
	NearBC,
	Pauktuuht,
	PHAC,
	Pimatisiwin,
	Population Health Improvement Research Network Library,
	New York Academy of Medicine,
	Health Quality Ontario,
	Health Research Council of New Zealand,
	ProQuest Conference Papers Index,
	Proquest Dissertation Abstracts,
	Rural and Remote Health
	The First Peoples Child and Family Review
	Tropical Disease Research Centre (CIET)
	UCLA Centre for Health Policy Research
	US National Library of Medicine
	WHO
	CAMH library
	OTRU library
	Google

The EMBASE search strategy is provided in Additional file 1 as an example of the search strategy used. In addition to our online search strategy, we contacted 22 researchers and Indigenous community-based groups in Canada and internationally. These contacts provided additional grey literature materials as well as further knowledge of ongoing tobacco control programs in Indigenous communities.

### Study selection

Each article (academic study or grey literature report) was assessed for inclusion on the following eligibility criteria. First, it must have been published or made available between 1994 and 2014. This 20 year period included the majority of tobacco control interventions. Second, the article must have addressed tobacco use in Indigenous communities. There were no geographic, gender or age restrictions. More specifically, the article must have involved either a majority Indigenous population or been statistically significant (for quantitative items) or adequate and meaningful (for qualitative items) to the Indigenous sample. Third, articles must have addressed interventions broadly defined (including provision of a health service or program, education or training program, media campaign or policy change) aimed at decreasing commercial tobacco use. Lastly, articles must have included an evaluative component of the intervention. Articles that only had a descriptive analysis were included if the intervention that was described was evaluated separately. There were no restrictions on research design or evaluation approach. Articles could use quantitative, qualitative or mixed methods approaches, and could include case control, cohort, cross-sectional, experimental, and intervention designs with no restrictions. Articles that were not available in English were excluded from the review.

**Fig. 1:**
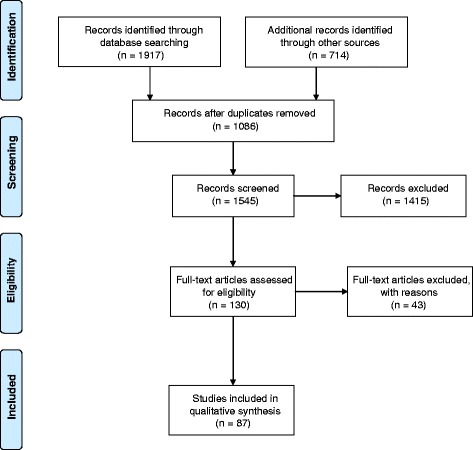
RETRAC search decision tree

Figure 1, the RETRAC search decision tree, presents a visual representation of our study selection process. The electronic peer reviewed database search and the grey literature search yielded 1917 and 714 records respectively. After duplicates were removed, 1545 records were screened for eligibility resulting in the identification of 130 articles of potential relevance to our review. Articles were screened using DistillerSR Systematic review software (© 2015 Systematic Review and Literature Review Software from Evidence Partners) which facilitates data extraction and analysis. Throughout the selection process, two members of the research team reviewed each article at risk of exclusion. Disagreements were resolved between the two reviewers by consensus. Articles were most typically excluded because they did not focus on Indigenous communities, lacked an intervention targeted at the reduction or prevention of commercial tobacco use and/or were missing an evaluation of the intervention.

Our search strategy included 87 articles. 65 were from peer-reviewed journals and 22 were from grey literature sources. 79 of the 87 articles provided one or more evaluations of a particular intervention. In total, the 79 articles provided 85 evaluations. The additional 8 (of 87) articles provided descriptive information about an intervention already included in the data-set. We used the descriptive accounts to enrich our understanding of how the intervention was implemented, who it worked for and why. In total, 93 studies (85 evaluations + 8 descriptive accounts) are represented in the data set. They are a combination of mixed method (25/93), quantitative (56/93) and qualitative (12/93) studies. The Additional file 2 provides a broad overview of the 93 studies included in this review detailing location and target population of the intervention as well as study design, sample size and outcomes.

### Appraising study quality

The quality appraisal tool (Additional file 3) was developed using components of a Public Health Agency of Canada Lessons Learned Data Extraction Guide, Kmet et al.’s standard quality assessment criteria, and the Well Living House quality assessment tool [16–18]. To accommodate the methodological heterogeneity of our data set, the tool was modified to include checklists for both qualitative and quantitative information. Quality was appraised along three elements: 1) rigour of evaluation methods, 2) strength of evidence and, 3) relevance to the Indigenous community. Each section had four questions of equal weight, aiding the researcher to determine whether the items had adequate evaluation methods related to design, implementation and analysis; adequate strength, including internal validity, external validity or triangulation and reflexivity; and a relevance to community, which assessed the studies’ alignment with community values, knowledge and priorities. The relevance to community section was included to explicitly rate the depth and incorporation of Indigenous perspectives and ways of knowing and doing in each intervention’s studies.

**Table 3:** Changes in Community Interest

		Activities															Outcome
		Individual					Community							Legislative			
Project Name	Study	Brief intervention	Pharmacotherapy	Behavioural support	Training health-care professionals	Incentives for Quitting	Media Campaigns	Education	Events	Distribution of Resources	Peer Support	Quitline/ Quit support	Ceremonial Practices	Smoking Ban	Sales Restrictions	Tax Increase	Community Interest
The Be Our Ally Beat Smoking (BOABS) study	Marley et al., 2014 [68] Marley et al., 2014 [27] Marley et al., 2014 [111]	✓	✓	✓			✓										↑
Deadly Choices	Malseed, 2013 [24] Malseed et al., 2014 [25]	✓	✓	✓				✓	✓			✓					↑
The Tobacco Action Project	Ivers, 2005 [22] Ivers et al., 2006 [31]	✓	✓		✓			✓	✓	✓				✓	✓		↑
The Tobacco Project	Thomas, Johnston & Fitz, 2010 [23]	✓	✓				✓	✓		✓			✓	✓	✓		↑
Top End Tobacco Project	Robertson et al., 2013 [44] Robertson, 2010 [28]	✓	✓					✓				✓		✓			↑
Wiidookowishin (Help Me) program	Bosma et al., 2014 [26] D’Silva et al., 2011 [58]		✓	✓				✓		✓							↑

↑: increase in outcome; ↓: decrease in outcome; No change: no change in outcome; ns: non-significant result

The quality appraisal tool was used to evaluate all 85 evaluations. For each article the tool generated a score between 0 and 1 and a resulting rating of weak (0-0.49), moderate (0.50-0.74) or strong (0.75-1.00). One of two reviewers independently reviewed each evaluation. Of the 85 program evaluations, 14 were scored strong, 44 as moderate, and 27 as weak. This review includes both strong and moderate studies and excludes weak studies.

### Inter-rater agreement testing

Interrater agreement was tested at both the midpoint and at the end of the quality assessment stage. At each stage, ten articles were randomly selected and the study quality of each was appraised independently by both reviewers. The intraclass correlation was used to assess inter-rater reliability, as is standard practice, and correlation coefficient values were interpreted as “>0.75 was excellent, 0.40–0.75 was fair to good and <0.40 was poor” [19]. In test one, the two reviewers showed good (ICC: 0.61) agreement on ten articles. In test two, the two reviewers again showed good (ICC: 0.69) agreement. In both cases when the apparent outlier was removed, we found excellent agreement (midpoint ICC 0.76, endpoint ICC 0.83) between the two reviewers for 90 % of the appraised articles.

### Data extraction

A data extraction form comprised of structured questions included: 13 questions about project characteristics (e.g. project goals, main activities, location, program site, program implementers and program users); 14 questions about the evidence base (e.g. study design, methodology, outcome measures, results and limitations); and five questions about learnings (e.g. strategies of success, cultural position of program, program successes and challenges). Three reviewers piloted the data extraction form with 13 studies. Following pilot testing, two reviewers independently completed data extraction on all remaining articles.

### Data synthesis

Narrative synthesis was chosen as the analytic method for this review because it is appropriate when synthesis of diverse evidence is needed [20]. A narrative synthesis is used to identify and textually describe meaningful patterns and themes in the included studies, synthesizing the evidence and noting variations in study characteristics. A meta-analysis or meta synthesis was precluded due to the diversity of study design and outcomes measures of the studies retrieved from the literature search.

## Results

The following sections detail the goals, location, population, activities, and nature of community engagement in the 73 interventions reported in this review. While 87 articles are included in the review, they represent a total of 73 interventions as certain interventions were the same across multiple studies.

The remaining discussion is organized around seven outcomes that were most frequently discussed in our dataset: community interest, knowledge, initiation, consumption, quit rates, smoke-free environments and prevalence. See Additional file 4 for a summary comparison of intervention characteristics and efficacy data for the seven outcomes.

### Description of interventions

This review includes 73 interventions that aimed to prevent (30), reduce (23) and/ or cease (42) the use of commercial tobacco; interventions that limited physical and social exposure (9); and interventions that limited access to and availability of commercial tobacco (2). Several studies addressed more than one of these aims.

The majority of interventions were located in Indigenous communities in the United States of America (25) and Australia (23). Additional interventions were in Canada (14), New Zealand (8), Fiji (1), Taiwan (1) and Australia and New-Zealand (1). A number of diverse Indigenous groups were represented in this literature set including Alaska Natives and people of Yup’ik ancestry, Native Hawaiians, Pacific Islanders, American Indians including members from the Ojibwe tribe, and Native Americans. Three political groups from Canada: First Nations, Inuit and Métis peoples participated in interventions. In Australia, Aboriginal and Torres Strait Islander people included Tiwi peoples, Jawoyn peoples, and Yolngu peoples. The Māori peoples of New Zealand were also engaged in interventions as well as ethnic Fijians from Fiji and the Aborigine population in Taiwan.

Intervention activities were grouped based on Ivers’ categorization as community level, individual level or legislative level [21]. Community level activities include education, media campaigns, quitlines and the use of cultural protocols or ceremonial practices; individual level activities include pharmacotherapy, behavioural support, training health professionals and incentives; legislative level activities refer to policies, laws and taxes. Fourteen interventions included individual activities, twenty-seven included community activities, and four included legislative activities. There were also interventions that included activities at multiple levels. Nineteen interventions used both individual and community level activities, two used community and legislative and seven interventions included individual, community and legislative level activities.

Interventions were organized and implemented by a number of different actors. Some interventions were organized and implemented entirely by Indigenous researchers, health professionals or community members while many others were implemented in partnership with Indigenous and non-Indigenous peoples. Interventions in this data-set were also implemented as mainstream health services. While this data was extracted from the literature, it was not possible to isolate the effect of these different categories on the seven outcomes analysed.

### Analysis of changes

Changes in outcomes were reported using quantitative and qualitative measures. The following results sections focus on those interventions that report either statistically significant change or qualitative results for each outcome.

#### Changes in community interest

Studies looked at three elements that we grouped together under a term called ‘community interest.’ These elements were: self-determination, local capacity, and the prioritization of tobacco. Seven studies representing six interventions reported that community interest improved as a result of a particular commercial tobacco control intervention (Table 3). Three studies were of strong quality, three studies were moderate and one was descriptive. Qualitative results in these studies demonstrate a greater sense of community interest to prioritize tobacco [22, 23] a feeling of greater self-determination to shape the health and well-being of both individuals and the community [24, 25] and development of local Indigenous capacity [22, 23, 26, 27].

**Table 4:** Changes in Knowledge

		Activities															Outcome
		Individual					Community							Legislative			
Project Name	Study	Brief intervention	Pharmacotherapy	Behavioural support	Training health-care professionals	Incentives for Quitting	Media Campaigns	Education	Events	Distribution of Resources	Peer Support	Quitline/ Quit support	Ceremonial Practices	Smoking Ban	Sales Restrictions	Tax Increase	Knowledge
The Boy and Woman Bear	Schinke, Moncher & Singer, 1994 [34]							✓									ns
Circles of Tobacco Wisdom	Nadeau et al., 2012 [33]							✓					✓				↑
Deadly Choices	Malseed, 2013 [24] Malseed et al., 2014 [25]	✓	✓	✓				✓	✓			✓					↑
FACETS curriculum	Schinke et al.,1996 [35]	✓	✓	✓		✓				✓							↑
Indigenous Smoke Free Project	Harvey et al., 2002 [37]						✓					✓					↑
Maningrida ‘Be Smoke Free’ Project	Johnston et al., 1998 [30]		✓	✓													↑
Native Comic Book Project	Montgomery et al., 2012 [32]							✓					✓				↑
No Smokes Project	Bell, 2012 [29]											✓					ns
Sacred Beginnings Project	Richards & Mousseau, 2012 [38]			✓				✓	✓	✓							ns
The Tobacco Action Project	Ivers, 2005 [22] Ivers et al., 2006 [31]	✓	✓		✓			✓	✓	✓				✓	✓		↑
Youth Action Alliance of Manitoulin Island	Irfan & Schwartz, 2012 [36]							✓	✓		✓		✓	✓		✓	↑

↑: increase in outcome; ↓: decrease in outcome; No change: no change in outcome; ns: non-significant result

For example, after three years of work in the Top-End of Australia’s Northern Territory and with support from the ‘Top-End Tobacco Project’ study investigators, local groups made tobacco a priority health issue in local health plans and agreements with all levels of government [23, 28]. Likewise, the ‘Be Our Ally Beat Smoking’ (BOABS) project in the Kimberley region of Western Australia attributed success to the “importance of local Aboriginal ownership, commitment, participation and control, [as well as] the flexibility to adapt interventions to local communities and circumstances, and taking sufficient time to allow this to occur” [27].

Common factors which contributed to a greater sense of community interest include: the presence of strong local drivers such as community leaders and council members, long-term investments in relationship building between community members and project staff, and the development of credibility and trust among project staff and community members.

#### Changes in knowledge

Thirteen studies, representing 11 interventions investigated changes in individual knowledge (Table 4). Three studies had strong quality and 10 studies had moderate. Five different types of knowledge were tested, including: the risks of and health conditions caused by smoking [22, 24, 25, 29–33] the traditional methods of using sacred tobacco [34, 35]; the causes of commercial tobacco misuse [36]; smoking cessation models [37]; and general knowledge of commercial tobacco [38]. Eight of the interventions revealed a positive impact on change in knowledge, while the effect of the other 3 interventions [29, 34, 38] is unknown due to insignificant results.

**Table 5:** Changes in Smoke-free Environments

		Activities															Outcome
		Individual					Community							Legislative			
Project Name	Study	Brief intervention	Pharmacotherapy	Behavioural support	Training health-care professionals	Incentives for Quitting	Media Campaigns	Education	Events	Distribution of Resources	Peer Support	Quitline/ Quit support	Ceremonial Practices	Smoking Ban	Sales Restrictions	Tax Increase	Smoke-free Environments
N/A	Walker et al., 2015 [45]	✓	✓	✓								✓					ns
Aboriginal Head Start Urban and Northern Communities Program	Mashford-Pringle, 2008 [42] Mashford-Pringle, 2012 [43]							✓		✓						✓	ns
Alaska Quitline	Boles et al., 2009 [40]		✓									✓					ns
Circles of Tobacco Wisdom	Nadeau et al., 2012 [33]							✓					✓				↑
Making Aboriginal Kids Walk Away (From Tobacco Abuse) (MAKWA)	Irfan, Schwartz & Bierre, 2012 [39]							✓	✓				✓	✓			↑
Maningrida ‘Be Smoke Free’ Project	Johnston et al., 1998 [30]		✓	✓													No change
Murri Places Smoke-free Spaces	Institute for Urban Indigenous Health, 2014 [41]	✓	✓	✓	✓			✓		✓				✓			ns
New Zealand’s Smoke-free Environments Amendment Act 2003 (SFEAA)	Watson et al., 2011 [46]													✓			ns
The Tobacco Action Project	Ivers, 2005 [22] Ivers et al., 2006 [31]	✓	✓		✓			✓	✓	✓				✓	✓		ns
Top End Tobacco Project	Robertson et al., 2013 [44] Robertson, 2010 [28]	✓	✓					✓				✓		✓			ns

↑: increase in outcome; ↓: decrease in outcome; No change: no change in outcome; ns: non-significant result

Three interventions produced a statistically significant increase in the knowledge of risks and health effects caused by smoking: Deadly Choices, the Tobacco Action Program and the FACETS curriculum. All of these interventions involved multiple components including school or community based education programs [22, 24, 25, 31, 35] sponsorship of cultural events [22, 24, 25, 31] and the use of ceremonial practices [22, 31, 35]. Moreover, all three interventions were implemented by Indigenous project officers [22, 31, 35] or Indigenous healthy lifestyle workers [24, 25]. For example, the Deadly Choices Program in South-East Queensland, Australia reported significant knowledge gains among 472 attendees. There was a 0.9 increase in mean knowledge scores among participants (from a pre-score of 7.9/12 to a post score of 8.8/12) after participation in health education activities [25]. Likewise, the Tobacco Action Project observed a 5 % (from 85 to 90 %) and 7 % (82 to 89 %) increase in the number of people (*n* = 351) who believed that tobacco use was linked to lung cancer and heart disease respectively [22, 31].

The FACETS curriculum based in five Native American communities in the Northeast, US was associated with a significant gain in comprehension of the use and importance of ceremonial tobacco. Of note, the program grounded its work in each community’s local culture using “native meals, story bags, sacred hoops and dance sticks” [35] as part of the curriculum. As a result of the program, a mean difference of 1.07 (mean, pre-test: 3.18 vs mean, post-test: 4.25) in the understanding of the use and cultural importance of tobacco was reported among individuals enrolled in the educational program.

Five studies showed a qualitative change in participants’ knowledge of the harmful effects of smoking [30, 32, 33]; benefits of smoking cessation [37] and the causes of commercial tobacco misuse [36]. Three [32, 33, 36] of the four interventions used community level activities such as education programs and ceremonial practices to influence change. These activities expanded knowledge among six [32] and 11 [36] youth respectively as well as 11 Elders [33]. For example, the Circle of Tobacco Wisdom program in a community in Minnesota, US, educated Elders about the health effects of tobacco and encouraged each Elder to share this knowledge with community members [33]. Similarly, the Maningrida ‘Be Smoke Free’ project in Australia used a school based education program to increase knowledge of the negative effects of smoking among 141 youth in two communities [30]. The Northern Queensland Tobacco Project was an individual level intervention which trained health-care professionals in tobacco brief intervention. Twenty-one Aboriginal health workers interviewed reported that the training offered opportunities to improve their knowledge on smoking cessation practice [37].

Several elements of program implementation were common to many of the eight interventions that reported positive change. First, three interventions [30, 32, 35, 38] provided access to culturally based health services and information by grounding its work in the particular local Indigenous context reflecting each community’s specific history, protocol and ceremonial framework. Second, five interventions engaged Aboriginal leadership leading to high levels of community ownership [22, 24, 25, 30, 31, 33, 36, 37].

#### Changes in smoke-free environments

Eleven studies investigated the development of smoke-free environments as an outcome (Table 5). These studies had either moderate (7/11) or strong quality (4/11) and described 10 interventions. Three studies report qualitative results [30, 33, 39] and the remaining eight report quantitative results. None of the results reported in the eight quantitative studies were statistically significant [22, 40–46].

**Table 6:** Changes in Initiation

		Activities															Outcome
		Individual					Community							Legislative			
Project Name	Study	Brief intervention	Pharmacotherapy	Behavioural support	Training health-care professionals	Incentives for Quitting	Media Campaigns	Education	Events	Distribution of Resources	Peer Support	Quitline/Quit support	Ceremonial Practices	Smoking Ban	Sales Restrictions	Tax Increase	Initiation
N/A	Schinke, Tepavac & Cole, 2000 [49]							✓		✓			✓				↑
Healthy Living in Two Worlds	Weaver & Jackson, 2010 [47]							✓					✓				↑
SmokingZine website	Bowen et al., 2012 [48]							✓									↓
The Tobacco Action Project	Ivers, 2005 [22] Ivers et al., 2006 [31]	✓	✓		✓			✓	✓	✓				✓	✓		ns

↑: increase in outcome; ↓: decrease in outcome; No change: no change in outcome; ns: non-significant result

The three qualitative studies represent three interventions of which two showed positive changes towards developing smoke-free environments while one did not. The two interventions that showed positive change either by establishing a smoke-free policy or through advocacy work, [33, 39] incorporated ceremonial practices in their educational programming. Making Aboriginal Kids Walk Away (From Tobacco Abuse) (MAKWA) intervention in Thunder Bay, Canada, educated 12 youth on the difference between ceremonial tobacco and commercial tobacco and the importance of smoke-free parks and beaches. This initiative included a powwow where these messages were reinforced. As a result of this intervention, a bylaw was ratified prohibiting tobacco use in parks and beaches, a tobacco-free sports program was established, and students were not allowed to use commercial tobacco while wearing school team uniforms or playing sports [39].

Both the MAKWA and Circles of Tobacco Wisdom interventions, which led to changes towards creating smoke-free environments, highlighted the importance of community involvement in program design and implementation [33, 39]. Irfan et al. explained that having an Aboriginal Youth Advisor implement the program led to a greater understanding of traditions and the community’s relationship with tobacco [39]. However, the Maningrida ‘Be Smoke Free’ Project, which did not lead to change, also involved community members in the creation and evaluation of the project [30].

#### Changes in initiation

Five studies with either moderate (2/5) or strong quality (3/5) investigated the age and rate of initiation of commercial tobacco use (Table 6). These five studies represented four different interventions. Only one of the studies was qualitative [47], while the other four were quantitative, two with a statistically significant outcome related to initiation [48, 49] and two without statistically significant results [22, 31].

**Table 7:** Changes in Consumption

		Activities															Outcome
		Individual					Community							Legislative			
Project Name	Study	Brief intervention	Pharmacotherapy	Behavioural support	Training health-care professionals	Incentives for Quitting	Media Campaigns	Education	Events	Distribution of Resources	Peer Support	Quitline/ Quit support	Ceremonial Practices	Smoking Ban	Sales Restrictions	Tax Increase	Consumption
N/A	Beckham et al., 2007 [60]			✓									✓				ns
N/A	Cowie, Glover & Gentles, 2014 [65]							✓									ns
N/A	Eades et al., 2012 [67]							✓					✓				ns
N/A	Gilligan, 2008 [57]		✓	✓	✓					✓	✓					✓	ns
N/A	Ivers et al., 2003 [63]		✓	✓													ns
N/A	Lin et al., 2013 [55]			✓						✓			✓				↓
N/A	Moncher & Schinke, 1994 [56]					✓											↓
N/A	Patten et al., 2013 [66]							✓									ns
N/A	Thomas et al., 2013 [50]	✓	✓		✓												no change
Aboriginal Head Start Urban and Northern Communities Program	Mashford-Pringle, 2008 [42] Mashford-Pringle, 2012 [43]							✓		✓						✓	ns
American Indian Not on Tobacco (N-O-T) program	Horn et al., 2005 [54] Horn et al., 2008 [81] Horn et al., 2009 [108]	✓		✓				✓		✓							↓
‘Bubblewrap’ campaign	Boyle et al., 2010 [59]					✓											ns
Canadian Quitlines	Hayward, Campbell & Sutherland-Brown, 2007 [62]											✓					ns
Circles of Tobacco Wisdom	Nadeau et al., 2012 [33]							✓					✓				↑↓
Deadly Choices	Malseed, 2013 [24] Malseed et al., 2014 [25]	✓	✓	✓				✓	✓			✓					ns
Healthy Living in Two Worlds	Weaver & Jackson, 2010 [47]							✓					✓				ns
Maningrida ‘Be Smoke Free’ Project	Johnston et al., 1998 [30]		✓	✓													no change
Métis Nation British Columbia’s Aboriginal ActNow BC Program	Wesche, Ryan & Carry, 2011 [64]			✓						✓			✓				ns
Murri Places Smoke-free Spaces	Institute for Urban Indigenous Health, 2014 [41]	✓	✓	✓	✓			✓		✓				✓			ns
New Zealand’s Smoke-free Environments Amendment Act 2003 (SFEAA)	Watson et al., 2011 [46]													✓			ns
Northern Queensland Indigenous Tobacco Project	Campbell et al., 2014 [53]	✓		✓				✓	✓					✓	✓		↓
Sacred Smoke	Aboriginal Cancer Care Unit, 2008 [51]	✓	✓	✓				✓									↓
SmokingZine website	Bowen et al., 2012 [48]							✓									ns
Think Smart	Johnson et al., 2009 [52]							✓									no change
The Tobacco Action Project	Ivers, 2005 [22] Ivers et al., 2006 [31]	✓	✓		✓			✓	✓	✓				✓	✓		↓
The Tobacco Project	Thomas, Johnston & Fitz, 2010 [23]	✓	✓				✓	✓		✓			✓	✓	✓		ns
Traditions of the Heart	Witmer et al., 2004 [61] Stefanich et al., 2005 [112]			✓				✓		✓							ns
Wiidookowishin (Help Me) program	Bosma et al., 2014 [26] D’Silva et al., 2011 [58]		✓	✓				✓		✓							ns

↑: increase in outcome; ↓: decrease in outcome; No change: no change in outcome; ns: non-significant resul

Two outcome measures expressed changes in initiation: 1. the proportion of participants who did not start using commercial tobacco during or after the intervention; 2. the proportion of participants who expressed no intention to start using commercial tobacco. Schinke et al.’s study [49] examined the first type of change, while Bowen et al. [48] examined the second. Weaver and Jackson examined both types of change [47].

Schinke et al. examined the initiation rate of 1,396 Native American youth in grades 3, 4 and 5 in five states in the US: North and South Dakota, Idaho, Montana, and Oklahoma. Two interventions were tested against a control group; one intervention called the “skills + community” intervention that included a culturally appropriate education program and distribution of resources among the community, and another intervention called the “skills-only” intervention that had only the culturally relevant education program. Across all three conditions initiation rates increased, however participants in the “skills only” intervention had the lowest initiation rates over the three years following the intervention (4.32 % pre to 10. 23 % post vs 5.16 % pre to 16.56 % post [“skills + community”] and 7.04 % pre to 17.83 % post [control group]). It should be noted that there was only a statistically significant change in the use of smokeless tobacco, not in the use of cigarettes [49]. The superiority of the “skills only” intervention was attributed to a concentrated approach of the education program while study authors argued that the effects of the “skills + community” may have been diluted throughout implementation [49].

Similar to the Schinke et al. “skills-only” intervention [49], the Healthy Living in Two Worlds intervention included an educational program for youth ages 9-13 in Buffalo, US that integrated ceremonial practice, such as Haudenosaunese smoke dance. However, nine of the 11 youth participating (81.8 %) had reported that they tried cigarette smoking after the intervention, while none of the youth reported ever trying cigarettes before the intervention. With regards to intention to start smoking, at pre and post-intervention, all the youth reported that they would refuse a cigarette if offered to them by a friend or family member [47]. The smoking prevention website intervention for youth ages 12-18 in South Dakota, US studied by Bowen et al. can also be seen as similar to the “skills-only” intervention, as this included only an educational website program that used culturally appropriate images and stories. This intervention led to a decline in intention to use commercial tobacco among 113 participants from 17 to 0 %, as compared to the control group which increased from 8 to 25 % [48].

#### Changes in consumption

Thirty-one studies observed the effect of 28 interventions on changes in the consumption of commercial tobacco (Table 7). These studies had either moderate (25/31) or strong (6/31) quality. Of the 31 studies, four had qualitative results [30, 33, 50, 51] and 27 had quantitative results, of which seven [22, 31, 52–56] are statistically significant and 20 [23, 24, 35, 41–43, 46–48, 57–67] are statistically non-significant.

**Table 8:** Changes in Quit Rates

		Activities															Outcome
		Individual					Community							Legislative			
Project Name	Study	Brief intervention	Pharmacotherapy	Behavioural support	Training health-care professionals	Incentives for Quitting	Media Campaigns	Education	Events	Distribution of Resources	Peer Support	Quitline/ Quit support	Ceremonial Practices	Smoking Ban	Sales Restrictions	Tax Increase	Quit Rates
N/A	Beckham et al., 2007 [60]			✓									✓				ns
N/A	Cowie, Glover & Gentles, 2014 [65]							✓									ns
N/A	DiGiacomo et al., 2007 [70]	✓	✓	✓													ns
N/A	Hensel et al., 1995 [73]						✓						✓				ns
N/A	Ivers et al., 2003 [63]		✓	✓													ns
N/A	Patten et al., 2014 [77]													✓			ns
Aboriginal Head Start Urban and Northern Communities Program	Mashford-Pringle, 2008 [42] Mashford-Pringle, 2012 [43]							✓		✓						✓	ns
Alaska Native Pregnant Women intervention	Patten et al., 2010 [76] Patten, 2012 [75]			✓						✓						✓	ns
Alaska Quitline	Boles et al., 2009 [40]		✓									✓					ns
American Indian Not on Tobacco (N-O-T) program	Horn et al., 2005 [54] Horn et al., 2008 [81] Horn et al., 2009 [108]	✓		✓				✓		✓							ns
The Be Our Ally Beat Smoking (BOABS) study	Marley et al., 2014 [68] Marley et al., 2014 [27] Marley et al., 2014 [111]	✓	✓	✓			✓										↑
Circles of Tobacco Wisdom	Nadeau et al., 2012 [33]							✓					✓				↑
Indigenous Smoke Free Project	Harvey et al., 2002 [37]						✓					✓					no change
Métis Nation British Columbia’s Aboriginal ActNow BC Program	Wesche, Ryan & Carry, 2011 [64]			✓						✓			✓				↑
New Zealand’s Smoke-free Environments Amendment Act 2003 (SFEAA)	Watson et al., 2011 [46]													✓			ns
PAU protocol	Santos et al., 2008 [71]		✓	✓													ns
Pregnant Aboriginal and Torres Strait Islander RCT	Gould & McEwan, 2012 [74]	✓	✓	✓	✓					✓	✓					✓	ns
Southcentral Foundation Tobacco Cessation Initiative	Fenn, Beiergrohslein & Ambrosio, 2007 [72]	✓	✓	✓													ns
STOMP: Stop Smoking by Mobile Phone	Bramley et al., 2005 [69]					✓		✓									↑
The Tobacco Action Project	Ivers, 2005 [22] Ivers et al., 2006 [31]	✓	✓		✓			✓	✓	✓				✓	✓		ns
Traditions of the Heart	Witmer et al., 2004 [61] Stefanich et al., 2005 [112]			✓				✓		✓							ns
Wiidookowishin (Help Me) program	Bosma et al., 2014 [26] D’Silva et al., 2011 [58]		✓	✓				✓		✓							ns

↑: increase in outcome; ↓: decrease in outcome; No change: no change in outcome; ns: non-significant result

Changes in consumption were measured by: tallying a change in daily smoking behaviours (the number of cigarettes or packs smoked per day); the heaviness of smoking index (HIS); levels of tobacco turnover (sales) within a community; or self-identification as a ‘current, former, or never smoker’.

Eight interventions reported either qualitative or statistically significant reductions in the consumption of commercial cigarettes. All eight interventions used a variety of activities to reduce the high rates of smoking in each community. Two interventions, the Tobacco Action Project and the Northern Queensland Indigenous Tobacco Project used activities at all three levels (individual, community and legislative), including brief intervention, school and community based education, and sales restrictions as well as smoke-free policies to promote the reduction of commercial tobacco use [22, 31, 53]. In eight communities in North Queensland, Australia where the Northern Queensland Indigenous Tobacco Project was implemented a significant difference of 16.8 % in the mean number of cigarettes smoked weekly among 449 people was revealed [53]. Two interventions [51, 54] used brief intervention and school based education to reduce cigarette consumption, while two other interventions used community based programs such as school and community education and the sponsorship of cultural or sporting events to reduce consumption rates [33, 54, 55]. For example, a six-hour preventative education seminar presented to urban dwelling Aborigines in Taiwan reduced their smoking behaviour at both immediate and four week follow-up with a significant mean difference in smoking behaviours between intervention participants (*n* = 64) and control group participants (*n* = 61) of 5.19 [55].

In contrast, three interventions had no measured effect on the consumption of commercial tobacco. Two of the three interventions used school and community education programs alone [30, 52] and reported that smoking behaviours remained constant before and after the intervention. For example, the school-based Think Smart curriculum used in rural-remote communities in Alaska, US had no effect on 30 day use of smokeless or cigarette tobacco among 652 youth who participated in the intervention [52]. The third intervention consisted of a legislative change in which a tax increase of 25 % was levied on commercial tobacco. According to Thomas et al. the tax increase, which was adopted in 18 communities throughout Central and Northern Australia, made no change in smoking or purchasing behaviour among community residents [50]. Lastly one intervention that also used school and community based education programs reported an increase in the use of commercial tobacco [33]. It was not possible to determine the effect of the culturally appropriate messaging provided in the education materials as this messaging was ubiquitous in interventions that reduced, increased and made no change on consumption rates.

#### Changes in quit rates

Twenty-three studies, representing 22 individual interventions, examined the effect of commercial tobacco control interventions on quit rates in Indigenous communities (Table 8). These 23 studies had either moderate (18) or strong (5) quality. Of the 23 studies, three reported qualitative results [33, 37, 64] and 20 reported quantitative results, in which two had statistically significant results related to this outcome [68, 69] and 18 had non-significant results [22, 40, 42, 46, 54, 58, 60, 61, 63, 65, 70–77]. Quit rates were calculated by the percentage of participants who quit using commercial tobacco, and were measured using self-report, carbon monoxide tests and/or cotinine tests.

**Table 9:** Changes in Prevalence

		Activities															Outcome
		Individual					Community							Legislative			
Project Name	Study	Brief intervention	Pharmacotherapy	Behavioural support	Training health-care professionals	Incentives for Quitting	Media Campaigns	Education	Events	Distribution of Resources	Peer Support	Quitline/ Quit support	Ceremonial Practices	Smoking Ban	Sales Restrictions	Tax Increase	Prevalence
N/A	Walker et al., 2015 [45]	✓	✓	✓								✓					ns
The Tobacco Action Project	Ivers, 2005 [22] Ivers et al., 2006 [31]	✓	✓		✓			✓	✓	✓				✓	✓		ns

↑: increase in outcome; ↓: decrease in outcome; No change: no change in outcome; ns: non-significant result

A total of five interventions showed a change in quit rates, four increased quits rates in their respective communities while one showed no change. Three of the four interventions that demonstrated a change in quit rates either incorporated ceremonial practices [33] or had culturally based activities [64, 69]. For example, for the STOMP project (Stop Smoking by Mobile Phone) in New Zealand, Māori researchers developed text messages that used Māori language and provided information on related Māori customs. The intervention led to a statistically significant change in quit rates between the intervention group and control group (total *n* = 1705): 26.1 % (intervention group) 11.2 % (control) at six weeks [69]. However the BOABS study which reported a statistically significant increase in quit rates between the intervention group (*n* = 163) and the usual care group (control group) based on cotinine test results did not include ceremonial practices or culturally appropriate activities. The same is true for the Indigenous Smoke Free Project that showed no change in quit rates among 24 participants. Both of these interventions only implemented brief intervention, NRT and counselling [37, 68].

### Changes in prevalence

Three studies [22, 31, 45] representing two interventions, investigated changes in smoking prevalence (the percentage of people who smoke tobacco cigarettes) as an outcome measure (Table 9). All three of these studies have strong quality. Neither The Tobacco Action Project [22, 31] nor a randomized controlled trial (RCT) of a second-hand-smoke intervention [45] produced a statistically significant change in prevalence.

The Tobacco Action Project was a five year community intervention that ran in remote areas of the Northern Territory of Australia. The program was implemented broadly at the community level with a myriad of interventions. The intervention components included: clinical cessation activities (brief intervention and NRT, community based educational campaigns, sponsorship of sporting and cultural events and adoption of smoke-free policies [22, 31]. Ivers et al. report a non-significant decrease of 1 percentage point in prevalence among 351 people interviewed in three intervention communities at baseline and follow-up. Prior to the intervention, prevalence was 68 %, while at the one-year follow-up prevalence was 67 % [31]. Nevertheless, Ivers et al. contend the project was a success due to the longevity of the relationships between community members and Aboriginal project officers. These long-term commitments allowed for the full involvement of community members in deciding intervention activities as well as adequate time to institute and observe community-wide changes.

In contrast, the three month second-hand smoke exposure reduction intervention implemented in Australia and New Zealand only used clinical cessation activities including brief intervention, NRT, individual counselling and referrals to national quitlines to reduce the prevalence of smoking among parents of young infants. Of the 293 participants contacted at 12-month follow-up, no significant change in smoking prevalence and intensity was observed. The study authors concluded that this intervention had no effect on prevalence [45].

## Discussion

Our investigation explored the effects of individual, community and legislative interventions on changes in the initiation, consumption and quit-rates of commercial tobacco use as well as changes in knowledge, prevalence, community interest and smoke-free environments. While the impact of these interventions was diverse, many led to desired changes in each of the outcomes, except in communities in Australia and New Zealand where prevalence rates were not impacted.

An important finding in our analysis was that education alone (either community or school based education) led to positive changes in reducing initiation rates, but did not have any measured effect on reducing consumption. This finding is consistent with Thomas et al’s. systematic review and meta-analysis of the effect of school-based tobacco prevention curricula where study authors found a 12 % reduction in initiation rates among child and adolescent never-smokers beyond a one-year follow-up [78]. In this review, interventions that incorporated educational programs with other activities such as pharmacotherapy and/or counselling did result in reductions. In accordance with the World Health Organization’s Framework Convention on Tobacco Control the use of comprehensive tobacco cessation strategies that include a myriad of activities such as taxation, smoke free policies, behavioural therapy and media campaigns are most effective at motivating and supporting people to quit [79].

The second aim of this review was to understand those interventions that produced community level change. Unfortunately, we found very little community level change. This may be due to the privileging of individual clinical activities in interventions rather than activities that reach the community more generally. It is also likely a reflection of the articles in our data set which primarily report on standard tobacco cessation and prevention indicators, which are individualistic in nature, rather than population measurements of change such as community censuses. Moreover, the lack of measured effect reported within many interventions in this dataset are explained by a variety of factors, including: poorly designed interventions, insufficient dosage and duration, incompatibility of intervention with community context, and study design flaws including small samples with insufficient power to detect small differences. Future research in this area should inform the development and evaluation of interventions by developing a set of indicators that reflect the breadth of meaningful change as defined by local Indigenous communities.

The third and fourth aim of this review was to uncover common elements that worked well across multiple interventions while exploring how programs and services reflect Aboriginal self-determination. To these aims, many of the interventions analysed in this review report high levels of community engagement and ownership. Interventions that led to positive changes were 1) led by Indigenous community members; 2) implemented in partnership with non-Indigenous health workers; 3) offered as mainstream health services. However, the importance of Indigenous led commercial tobacco control interventions is three-fold. First, it demonstrates a growing recognition of the need to address the high rates of smoking in Indigenous communities. Second, it recognizes that interventions will have greater community relevance if programs are supported and rooted in local community context. Third, it reflects the growing demand, made by Aboriginal communities, for control over health services through the full participation of Aboriginal individuals in decision making and implementation.

Further, consistent with approaches to Indigenous health knowledge translation, this review found a preference for ‘within the community’ messages [80]. These health messages incorporate culturally appropriate icons and symbols and are relayed by members of the community. This was made evident through the frequent use of culturally relevant health promotion materials that were adapted to reflect the communities’ unique history and culture as well as the use of Aboriginal project staff and research officers in many of the interventions. Moreover, the 73 interventions described in this review were found to be most effective when local protocols were acknowledged and ceremony adhered. For example, many program organizers relied on the guidance and knowledge of community Elders in creating and implementing their intervention. This relationship was found to be an important way to honour local protocols and Aboriginal self-determination.

A number of challenges concerning recruitment, funding, and intervention fidelity were reported across multiple interventions. A lack of dedicated or delayed funding undermined the success of three interventions by delaying activities [22, 31] and limiting human resource capacity to manage the project [23, 81]. Recruitment and retention of participants was also a challenge for many interventions. Travel constraints and the distance to intervention sites impacted the number of participants recruited in five programs [22, 27, 31, 35, 36, 39]. A lack of interest [24, 66] concerns about stigma and onerous consent processes [39] were identified as challenges in three other interventions.

Interventions were seldom delivered as intended. Nine interventions [22, 24, 25, 31, 37, 45, 53, 57, 67, 74, 82] were unable to mainstream intervention procedures into clinical or community practice. For three interventions, staff turnover led to difficulties in ensuring proper adherence to intervention components, whereas time constraints and competing health priorities led to low levels of implementation fidelity among project officers implementing the Tobacco Action Project and the Northern Queensland Indigenous Tobacco Project [22, 31, 53]. Harvey et al. in their evaluation of the Indigenous Smoke Free Project attributed this low intervention fidelity to an inconsistency with ‘Murri way” [37]. While a lack of intervention fidelity was considered a challenge by many evaluators, it can also be seen as an act of self-determination. In an earlier study of Indigenous knowledge translation of three Indigenous communities in Canada, Smylie et al. reported that interventions were found to be the least effective when they did not reflect and build on locally specific experiences and community-generated knowledge [80]. In this review, interventions were not always grounded in the local experience. Low intervention fidelity may actually reflect an act of self-determination made by local Indigenous experts who implement components of the intervention that best mirror their own local context. This approach reflects a commitment to the rights of Aboriginal peoples to choose their practices and sees intervention organizers as facilitators of this change [83].

Ultimately, it appears that not one kind of intervention will lead to positive changes in reductions in and protection from commercial tobacco use in Indigenous communities. Rather there are a number of common elements or strategies that work well to produce change. These strategies include cultivating meaningful relationships with community members, providing access to culturally based health care, and engaging with and grounding work in cultural protocol and practice. Future interventions should ensure that their work is multi-faceted, rooted in Indigenous ways of knowing and doing, and deferential to the right of Aboriginal self-determination.

### Limitations

While three other systematic reviews have investigated interventions that reduce the use of commercial tobacco in Indigenous communities, this review addressed a research gap by incorporating a greater breadth of information from the US and Canada. To that end, our grey literature search focused primarily on North American Indigenous groups and contacts. A second limitation relates to time and resources. Due to the number of references identified, only one reviewer completed data extraction and quality assessment for each article. To address this limitation, three rounds of pilot testing and an inter-rater reliability test was completed by the two reviewers. Third our data set may be limited by a publication bias as our understanding of this research was restricted to the available literature, although we did contact Indigenous researchers to solicit unpublished work. Fourth there was a paucity of community relevant evaluations that were included in the data set. Fifth although most interventions occurred after 1994, narrowing the search to the past 20 years might have eliminated useful work.

## Conclusion

In the process of this review, we investigated 73 commercial tobacco control interventions in Indigenous communities globally. These interventions incorporated a myriad of activities to reduce, cease or protect Indigenous peoples from the harms of commercial tobacco use. Our analysis focused on the effects of these interventions on changes in initiation, knowledge, smoke-free environments, consumption, quit rates, prevalence and community interest. The extent of this research in Indigenous communities globally suggests a growing prioritization and readiness to address the high rates of commercial tobacco use through both comprehensive and tailored interventions.

Our systematic review provides a meaningful investigation of the approaches and qualities of commercial tobacco control and prevention programs that have been implemented and found success in Indigenous communities globally. Overall, it appears that there is not one type of intervention nor a combination of activities that will most likely support the reduction of commercial tobacco use in Indigenous communities but rather programs that 1) Use a comprehensive approach inclusive of multiple activities, 2) Centre Aboriginal leadership, 3) Make long term community investments, and 4) Provide culturally appropriate health materials and activities produce desired changes.

## Additional files

Additional file 1: **Search Strategy. Example of the studies search strategy using the EMBASE database.** (PDF 53 kb)

Additional file 2: **Intervention Characteristics.** Description of data: A table detailing the location, population, length and activities of each intervention as well as study design, sample size, outcomes and quality score [22–77, 81, 82, 84–112]. (PDF 296 kb)

Additional file 3: **Quality Assessment Tool.** (PDF 71 kb)

Additional file 4: **Title of data: Intervention Activity and Outcome Efficacy Data.** Description of data: Summary comparison of intervention characteristics and efficacy data for the seven outcomes. (XLSX 18 kb)

## Abbreviations

NRT: nicotine replacement therapy
US: United States of America

## Footnotes

1 The term Indigenous is used to describe the global and international context. In Canada, the term Aboriginal is used to refer to First Nations, Métis and Inuit populations.
