# Supplementary material for: Effective strategies to reduce commercial tobacco use in Indigenous communities globally: A systematic review
Source: BMC Public Health. 2016 Jan 11;16:21. doi: 10.1186/s12889-015-2645-x (PMC4710008; doi:10.1186/s12889-015-2645-x)
Supplement: Supplementary file 1 — Search Strategy. Example of the studies search strategy using the EMBASE database. (PDF 53 kb) [file 12889_2015_2645_MOESM1_ESM.pdf]

Additional File 1: Search Strategy
Database: Embase <1980 to 2014 Week 45>
----
1 exp indigenous people/ or exp American Indian/ or exp Aborigine/ (15607)
2 exp Eskimo/ (2597)
3 exp Inuit/ (86)
4 exp Metis/ (9)
5 (aborigin* or native or "north american indian" or inuit or inuk or "first nations" or "native canadian" or "native american").mp. [mp=title, abstract, subject headings, heading word, drug trade name, original title, device manufacturer, drug manufacturer, device trade name, keyword] (177628)
6 (maori or "pacific islander" or "native Alaska*" or "alaska Native*" or "native hawaiian*" or "torres strait islander*" or yupik or aleut).mp. [mp=title, abstract, subject headings, heading word, drug trade name, original title, device manufacturer, drug manufacturer, device trade name, keyword] (8191)
7 1 or 2 or 3 or 4 or 5 or 6 (197602)
8 exp paternal smoking/ or exp "smoking and smoking related phenomena"/ or exp parental smoking/ or exp adolescent smoking/ or exp maternal smoking/ or exp smoking/ or exp smoking habit/ or exp passive smoking/ (214244)
9 exp tobacco dependence/ or exp "tobacco use"/ or exp tobacco consumption/ (210987)
10 exp smoking cessation program/ or exp smoking ban/ or exp smoking cessation/ or exp smoking regulation/ (39557)
11 ("smoking prevention" or "tobacco control" or "smoking reduction" or intervention or program or initiative or "program evaluation" or "tobacco reduction").mp. [mp=title, abstract, subject headings, heading word, drug trade name, original title, device manufacturer, drug manufacturer, device trade name, keyword] (1121907)
12 (tax* or "smoking restriction*" or "tobacco reduction strateg*" or "tobacco control strateg*" or "quit smoking").mp. [mp=title, abstract, subject headings, heading word, drug trade name, original title, device manufacturer, drug manufacturer, device trade name, keyword] (133978)
13 8 or 9 (222994)
14 10 or 11 or 12 (1270799)
15 7 and 13 and 14 (607)
16 limit 15 to yr="1994 - 2014" (595)
17 Selected references (313)
