# Supplementary material for: Effective strategies to reduce commercial tobacco use in Indigenous communities globally: A systematic review
Source: BMC Public Health. 2016 Jan 11;16:21. doi: 10.1186/s12889-015-2645-x (PMC4710008; doi:10.1186/s12889-015-2645-x)
Supplement: Supplementary file 2 — Intervention Characteristics. Description of data: A table detailing the location, population, length and activities of each intervention as well as study design, sample size, outcomes and quality score [22–77, 81, 82, 84–112]. (PDF 296 kb) [file 12889_2015_2645_MOESM2_ESM.pdf]

Additional File 2: Intervention Characteristics

Study	Project Name	Location	Ethnicity	Intervention Length	Main Activities	Study Design	Sample Size	Outcomes Measured	Study Quality
Aboriginal Cancer Care Unit, 2008 [49]	Sacred Smoke	Canada	Aboriginal	8 weeks	Brief intervention<br>Pharmacotherapy<br>Behavioural support<br>Education	Before and After	*n* = 20 (at baseline and follow-up)	Consumption	Moderate
Aboriginal Cancer Care Unit, 2008 [49]	Anishnawbe Mushkiki – Sema Kenjigewin Aboriginal Tobacco Misuse Program	Canada	Aboriginal	12 weeks	Behavioural support<br>Incentives for quitting	Before and After	*n* = 6 (baseline), 3 (follow-up)	Quit rates	Weak
Adams and Briggs, 2005 [82]	Koori Tobacco Cessation Project	Australia	Aboriginal	3-5 years	Pharmacotherapy<br>Training healthcare professionals<br>Behavioural support<br>Distribution of resources	Before and After	*n* = 115 (baseline), 36 (immediate follow-up) 15 (3 month follow-up)	Consumption, Quit rates	Weak
Adams and Briggs, 2005 [82]	No more Bundah, Winnunga Nimmityjah,	Australia	Aboriginal	3-5 years	Pharmacotherapy<br>Behavioural support<br>Education	Before and After	*n* = 30 (baseline and follow-up)	Quit rates	Weak
Adams and Briggs, 2005 [82]	Rumbalara Football Netball Club, Tobacco Project	Australia	Koori population	7 months	Education<br>Events<br>Distribution of resources	Cross-sectional	*n* = 25	Attitudes	Weak
Beckham et al., 2007 [58]	N/A	USA	Native Hawaiian	3 years	Behavioural support<br>Traditional rituals	Before and After	*n* = 209 (baseline), 177 (follow-up)	Consumption<br>Quit rates	Moderate

Study	Project Name	Location	Ethnicity	Intervention Length	Main Activities	Study Design	Sample Size	Outcomes Measured	Study Quality
Bell, 2012 [27]	No Smokes Project	Australia	Indigenous	1-3 hours	Media campaigns	Cross-sectional	*n* = 67	Knowledge	Moderate
Boles et al., 2009 [38]	Alaska Quitline	USA	ANAI (Alaska Native/American Indians)	5 months	Pharmacotherapy<br>Quitline	Cross-sectional	*n* = 112	Smoke-free environments<br>Quit rates	Moderate
Bosma et al., 2014 [24]	Wiidookowishin (Help Me) program	USA	American Indian	1 month	Pharmacotherapy<br>Behavioural support<br>Education<br>Distribution of resources	Cross-sectional	*n* = 20	Community Interest	Strong
Bowen et al., 2012 [46]	SmokingZine website	USA	American Indians	1 month	Education	RCT	Intervention: *n* = 113 (baseline)<br>*n* = 102 (follow-up)<br>Control group: yes	Initiation<br>Consumption	Moderate
Boyle et al., 2010 [57]	'Bubblewrap' campaign	Australia	Aboriginal	7 weeks	Media campaigns	Cross-sectional	*n* = 198	Consumption	Moderate

Study	Project Name	Location	Ethnicity	Intervention Length	Main Activities	Study Design	Sample Size	Outcomes Measured	Study Quality
Bramley et al., 2005 [67]	STOMP: Stop Smoking by Mobile Phone	New Zealand	Maori	4-6 months	Media campaigns<br>Education	RCT	Intervention: *n* = 176 (baseline)<br>*n* = 160 (6 week follow-up)<br>*n* = 80 (26 week follow-up)<br>Control group: yes	Quit rates	Moderate
Burgess, 2008 [93]	Maningrida ‘Smokebusters’ campaign	Australia	Aboriginal	Not specified	Behavioural support<br>Education<br>Distribution of resources	Before and After	Not reported	Consumption<br>Knowledge	Weak
Campbell et al., 2014 [51]	Northern Queensland Indigenous Tobacco Project	Australia	Aboriginal and Torres Strait Islander	Not specified	Brief intervention<br>Behavioural support<br>Education<br>Events<br>Sales restrictions<br>Smoking ban	RCT	Intervention: *n = 449*<br>Control: *n* = 253	Consumption	Moderate
Centre for Excellence in Indigenous Tobacco Control, 2007 [83]	BUTT OUT	Australia	Indigenous	2 years	Pharmacotherapy<br>Behavioural support	Observational	*n* = 64 (baseline), 26 (follow-up)	Quit rates	Weak
Chansonneuve, 2007 [84]	Sacred Smoke	Canada	Aboriginal	8 weeks	Education	Not reported	Not reported	Consumption	Weak

Study	Project Name	Location	Ethnicity	Intervention Length	Main Activities	Study Design	Sample Size	Outcomes Measured	Study Quality
Cosh et al., 2013 [85]	N/A	Australia	Indigenous Australians	1 year	Quitline	Before and After	*n* = 9928 (baseline), 6198 (3 month follow-up)	Quit rates	Weak
Cowie, Glover & Gentles, 2014 [63]	N/A	New Zealand	Maori and Pacific Island ethnic groups	1-2 year	Education	Cross-sectional	*n* = 428	Consumption<br>Quit rates	Moderate
D'Silva et al., 2011 [56]	Wiidookaawishin Help Me Program	USA	American Indian	1-3 months	Pharmacotherapy<br>Behavioural support<br>Education<br>Distribution of resources	Before and After	*n* = 141 (baseline and follow-up)	Consumption<br>Quit rates	Moderate
Daley et al., 2010 [86]	All Nations Breath Of Life	USA	American Indian	12 weeks	Pharmacotherapy<br>Behavioural support<br>Incentives for quitting<br>Education<br>Distribution of resources	CBR	*n = 108*	Quit rates	Weak
Department of Health, Australia Government, 2013 [87]	Breaking the Chain	Australia	Indigenous Australians	10 weeks	Pharmacotherapy<br>Quit support for health professionals<br>Education<br>Smoking ban	Cross-sectional	*n = 350*	Knowledge	Weak
DiGiacomo et al., 2007 [68]	N/A	Australia	Indigenous Australians (Aboriginal and Torres Strait Islander people)	10 months	Brief intervention<br>Pharmacotherapy<br>Behavioural support	Before and After	*n* = 37 (baseline), 32 (follow-up)	Quit rates	Moderate

Study	Project Name	Location	Ethnicity	Intervention Length	Main Activities	Study Design	Sample Size	Outcomes Measured	Study Quality
Eades et al., 2012 [65]	N/A	Australia	Aboriginal and Torres Strait Islander	4.5 years	Education<br>Traditional rituals	RCT	Intervention: n = 148<br>Control: n = 115	Consumption	Moderate
Fenn, Beiergrohslein & Ambrosio, 2007 [70]	Southcentral Foundation Tobacco Cessation Initiative	USA	Alaska Native	1 year	Brief intervention<br>Pharmacotherapy<br>Behavioural support	Cross-sectional	n = 902	Quit rates	Moderate
Gilligan, 2008 [55]	N/A	Australia	Aboriginal and Torres Strait Islander people	1-2 year	Pharmacotherapy<br>Training health-care professionals<br>Behavioural support<br>Distribution of resources	RCT	Intervention: n = 76<br>Control: n = 55	Consumption	Strong
Glover et al., 2014 [88]	N/A	New Zealand	Maori	8 weeks	Events	RCT	Intervention: n = 16<br>Control: n = 8	Consumption<br>Quit rates	Weak
Gould & McEwan, 2012 [72]	Pregnant Aboriginal and Torres Strait Islander RCT	Australia	Aboriginal and Torres Strait Islander	4.5 years	Brief intervention<br>Pharmacotherapy<br>Training health-care professionals<br>Behavioural support<br>Peer support<br>Distribution of resources	RCT	Intervention: n = 148<br>Control: n = 115	Quit rates	Moderate
Government of Victoria, 2014 [89]	Victorian Closing the Gap in Health Outcomes Initiative	Australia	Aboriginal People	4 years	Quitline	Cross-sectional	n = 313	Consumption	Weak

Study	Project Name	Location	Ethnicity	Intervention Length	Main Activities	Study Design	Sample Size	Outcomes Measured	Study Quality
Grigg, Waa & Bradbrook, 2008 [90]	The 'It's about whānau' (IAW) campaign	New Zealand	Maori	4-6 months	Media campaigns<br>Quitline	Before and After	*n* = 473 (baseline), 655 (follow-up)	Use of quitline	Moderate
Groth-Marnat, Leslie & Renneker, 1996 [91]	N/A	Fiji	"ethnic Fijans"	21 months	Pharmacotherapy<br>Behavioural support	Cross-sectional	*n* = 147	Consumption	Weak
Harvey et al., 2002 [35]	Indigenous Smoke Free Project	Australia	Indigenous	1 year	Media campaigns<br>Quitline	Before and After	*n* = 34 (baseline), 21 (follow-up)	Knowledge<br>Quit rates	Moderate
Hayward, Campbell & Sutherland-Brown, 2007 [60]	Canadian Quitlines	Canada	Aboriginal	4.5 years	Quitline	Before and After	*n* = 516 (baseline), 321 (6 month follow-up)	Consumption	Moderate
Hensel et al., 1995 [71]	N/A	USA	Alaska Native	14 months	Media campaigns<br>Traditional rituals	Before and After	*n* = 193 (baseline)<br>*n* = 156 (3 mth follow-up)<br>*n* = 71 (6 mth follow-up)<br>*n* = 55 (9 mth follow-up)	Quit rates	Moderate

Study	Project Name	Location	Ethnicity	Intervention Length	Main Activities	Study Design	Sample Size	Outcomes Measured	Study Quality
							n = 19 (1 year follow-up)		
Hiscock et al., 2009 [92]	PEGS: Preparation, Education, Giving up and Staying Smoke Free Programme	New Zealand	Maori	Not specified	Education<br>Traditional rituals	Before and After	*n* = 11, 325 (baseline), 7778 (follow-up)	Quit rates	Weak
Holt et al., 2005 [94]	N/A	New Zealand	Maori	7 weeks	Pharmacotherapy<br>Behavioural support<br>Distribution of resources	RCT	Intervention: *n* = 88<br>Control: *n* = 46	Quit rates	Weak
Horn et al., 2005 [52]	American Indian Not on Tobacco (N-O-T) program	USA	American Indian	4-6 months	Brief intervention<br>Education<br>Distribution of resources	Non RCT	Intervention: *n* = 54<br>Control: *n* = 20	Consumption<br>Quit rates	Strong
Institute for Urban Indigenous Health, 2014 [95]	Deadly Choices	Australia	Aboriginal and Torres Strait Islander	Not specified	Brief intervention<br>Pharmacotherapy<br>Behavioural support	Before and After	Not reported	Consumption	Weak

Study	Project Name	Location	Ethnicity	Intervention Length	Main Activities	Study Design	Sample Size	Outcomes Measured	Study Quality
Institute for Urban Indigenous Health, 2014 [39]	Murri Places Smoke-free Spaces	Australia	Aboriginal and Torres Strait Islander	2.5 year	Brief intervention<br>Pharmacotherapy<br>Training health-care professionals<br>Behavioural support<br>Education<br>Distribution of resources<br>Smoking ban	Cohort Study	*n* = 391	Consumption<br>Smoke-free environments	Strong
Inuit Tapiriit Kanatami, 2011 [96]	Blue Light Campaign	Canada	Inuit	Not specified	Media campaigns<br>Education<br>Events	Before and After	*n* = 108 (baseline and follow-up)	Consumption<br>Smoke-free environments	Weak
Inuit Tobacco Free Network, 2011 [97]	Smoke Stories: Quit Clips by Inuit Youth — Video Screening Contest	Canada	Inuit	Not specified	Education	Cross-sectional	*n* = 37	Knowledge	Weak
Irfan & Schwartz, 2012[34]	Youth Action Alliance of Manitoulin Island	Canada	Aboriginal	Not specified	Peer support<br>Education<br>Events<br>Traditional rituals<br>Smoking bans	Cross-sectional	*n = 12*	Knowledge	Moderate
Irfan, Schwartz & Bierre, 2012 [37]	Making Aboriginal Kids Walk Away (From Tobacco Abuse) (MAKWA)	Canada	Aboriginal/ First Nations	Not specified	Education<br>Events<br>Traditional rituals<br>Smoking bans	Cross-sectional	*n = 12*	Smoke-free environments	Moderate

Study	Project Name	Location	Ethnicity	Intervention Length	Main Activities	Study Design	Sample Size	Outcomes Measured	Study Quality
Ivers et al., 2003 [61]	N/A	Australia	Indigenous	6 months	Pharmacotherapy<br>Behavioural support	Before and After	*n* = 40 (baseline), 34 (follow-up)<br>Control Group: yes	Consumption<br>Quit rates	Moderate
Ivers, 2005 [20]	The Tobacco Action Project	Australia	Tiwi people, Jawoyn people, Yolngu people	1-2 year	Training health care professionals<br>Education<br>Events<br>Distribution of resources<br>Smoking ban	Before and After	*n* = 72 (baseline), 71 (follow-up)	Community interest<br>Initiation<br>Knowledge<br>Consumption<br>Smoke-free environments<br>Quit rates<br>Prevalence	Strong
Ivers et al., 2005 [98]	The Tobacco Project	Australia	Aboriginal (91%)	1 year	Media campaigns	Before and After	*n* = 351 (baseline and follow-up)	Consumption<br>Quit rates	Moderate
Ivers et al., 2006 [29]	The Tobacco Action Project	Australia	Aboriginal	1 year	Brief intervention<br>Pharmacotherapy<br>Training health-care professionals Education<br>Events<br>Distribution of resources<br>Sales restrictions<br>Smoking ban	Before and After	*n* = 643 (baseline), 628 (follow-up)	Knowledge<br>Initiation<br>Consumption<br>Prevalence	Strong

Study	Project Name	Location	Ethnicity	Intervention Length	Main Activities	Study Design	Sample Size	Outcomes Measured	Study Quality
Ivers et al., 2006 [99]	The Tobacco Project	Australia	Not reported	2 year	Behavioural support<br>Distribution of resources<br>Traditional rituals	Before and After	*n* = 29 (baseline), 25 (follow-up)	Compliance with regulations	Moderate
Johnson et al., 1997 [80]	GAINS: Giving American Indians No-smoking Strategies Project	USA	American Indians.	1 year	Brief intervention<br>Training health care professionals<br>Behavioural support	Non RCT	Intervention: *n* = 302<br>Control: *n* = 209	Knowledge<br>Quit rates	Weak
Johnson et al., 2009 [50]	Think Smart	USA	Native Alaskans	4-6 months	Education	RCT	Intervention: *n* = 652<br>Control: *n* = 637	Consumption	Moderate
Johnston et al., 1998 [28]	Maningrida 'Be Smoke Free' Project	Australia	Aboriginal	2 weeks	Pharmacotherapy<br>Behavioural support	Non RCT	Intervention: *n* = 151 (baseline)<br>*n* = 118 (follow-up)<br>Control Group: yes	Knowledge<br>Consumption<br>Smoke-free environments	Moderate
Lin et al., 2013 [53]	N/A	Taiwan	Aborigines	6 hours	Behavioural support<br>Distribution of resources<br>Traditional rituals	RCT	Intervention: *n* = 64<br>Control: *n* = 61	Consumption	Moderate

Study	Project Name	Location	Ethnicity	Intervention Length	Main Activities	Study Design	Sample Size	Outcomes Measured	Study Quality
Malseed, 2013 [22]	Deadly Choices	Australia	Aboriginal and Torres Strait Islanders	7 weeks	Education<br>Events	Before and After	School:<br>Intervention: *n* = 65<br>Control: *n* = 16<br><br>Community: *n* = 479 (baseline and follow-up)	Community interest<br>Knowledge<br>Consumption	Moderate
Malseed et al., 2014 [23]	Deadly Choices	Australia	Aboriginal and Torres Strait Islander	Not specified	Brief intervention<br>Pharmacotherapy<br>Behavioural support<br>Quitline	Non RCT	Intervention: *n* = 472	Community interest<br>Knowledge	Moderate
Marley et al., 2014 [66]	The Be Our Ally Beat Smoking (BOABS) study	Australia	Aboriginal	Not specified	Media campaigns	CBR	Not reported	Quit Rates	Moderate
Marley et al., 2014 [25]	The Be Our Ally Beat Smoking (BOABS) study	Australia	Aboriginal and Torres Strait Islander people	1 year	Brief intervention<br>Pharmacotherapy<br>Behavioural support	RCT	Intervention: *n* = 163 (baseline)<br>*n* = 144 (12 mth follow-up)<br>Control Group: yes	Community interest	Strong

Study	Project Name	Location	Ethnicity	Intervention Length	Main Activities	Study Design	Sample Size	Outcomes Measured	Study Quality
Mashford-Pringle, 2008 [40]	Aboriginal Head Start Urban and Northern Communities Program	Canada	Aboriginal - First Nations "status" and "non-status" people as well as Métis peoples	10 months	Tax increase	Before and After	*n* = 29 (baseline and follow-up)	Consumption<br>Smoke-free environments<br>Quit rates	Strong
Mashford-Pringle, 2012 [41]	Aboriginal Head Start Urban and Northern Communities Program	Canada	Aboriginal - from all 3 groups, but mostly First Nations and Metis	10 months	Education<br>Distribution of resources	Before and After	*n* = 44 (baseline), 29 (follow-up)	Consumption<br>Smoke-free environments	Moderate
McKennitt & Currie, 2012 [100]	No name	Canada	Aboriginal	60 minutes	Education	RCT	Intervention: *n* = 11<br>Control: *n* = 7	Initiation<br>Knowledge	Weak
Mitchell, 2007 [101]	Aniqsaattiarniq – Breathing Easy	Canada	Inuit	Not specified	Training healthcare professionals Education<br>Distribution of resources	Before and After	Nursing Stations: *n* = 63 (baseline), 24 (follow-up)<br><br>School contacts: *n* = 62 (baseline), 5(follow-up)	Smoke-free environments	Weak

Study	Project Name	Location	Ethnicity	Intervention Length	Main Activities	Study Design	Sample Size	Outcomes Measured	Study Quality
Moncher & Schinke, 1994 [54]	N/A	USA	Native American/ American Indian	4-6 months	Incentives for quitting	RCT	Intervention: *n* = 1,386 Control Group: yes	Consumption	Moderate
Montgomery et al., 2012 [30]	Native Comic Book Project	USA	American Indians and Alaska Natives	8 weeks	Education<br>Traditional rituals	Before and After	*n* = 6 (baseline and follow-up)	Knowledge	Moderate
Nadeau et al., 2012 [31]	Circles of Tobacco Wisdom	USA	American Indian	1 year	Education<br>Traditional rituals	Before and After	*n* = 13 (baseline), 12 (follow-up)	Knowledge<br>Consumption<br>Smoke-free environments<br>Quit rates	Moderate
Patten et al., 2010 [74]	Patten Alaska Native Pregnant Women intervention	USA	Alaska Native - Yupik ethnicity	7 months - 11 months	Tax increase	RCT	Intervention: *n* = 17 Control: *n* = 18	Quit rates	Strong
Patten, 2012 [73]	Patten Alaska Native Pregnant Women intervention	USA	Alaska Native - Yup'ik or Cup'ik ethnicity	7 months - 11 months	Behavioural support<br>Distribution of resources	RCT	Intervention: *n* = 39 Control: *n* = Not reported	Quit rates	Moderate

Study	Project Name	Location	Ethnicity	Intervention Length	Main Activities	Study Design	Sample Size	Outcomes Measured	Study Quality
Patten et al., 2013 [64]	N/A	USA	Alaska Native	3 days	Education	Before and After	*n* = 21 (baseline), 13 (follow-up)	Consumption	Moderate
Patten et al., 2014 [75]	N/A	USA	Alaska Native	5 weeks	Smoking ban	RCT	Intervention: *n* = 41 *n* = 38 (6 week follow-up) *n* = 41 (6 mth follow-up) Control: *n* = 27	Quit rates	Strong
Richards & Mousseau, 2012 [36]	Sacred Beginnings Project	USA	American Indian	6 weeks	Behavioural support<br>Education<br>Events<br>Distribution of resources	CBR	Intervention: *n* = 39 (baseline) *n* = 28 (follow-up) Control Group: yes	Knowledge	Strong
Robertson et al., 2013 [42]	Top End Tobacco Project	Australia	Aboriginal/ Indigenous	5 years	Smoking ban	Observationa l	*n* = 3	Smoke-free environments	Strong
Santos et al., 2008 [69]	PAU protocol	USA	Native Hawaiians	1-2 year	Pharmacotherapy<br>Behavioural support	Cross-sectional	*n* = 150	Quit rates	Moderate

Study	Project Name	Location	Ethnicity	Intervention Length	Main Activities	Study Design	Sample Size	Outcomes Measured	Study Quality
Schinke, Moncher & Singer, 1994 [32]	The Boy and Woman Bear	USA	American Indian or Native American.	less than one month	Education	RCT	Intervention: *n* = 233<br>Control: *n* = 135	Knowledge	Moderate
Schinke et al.,1996 [33]	FACETS curriculum	USA	Native American	4 months	Brief intervention<br>Pharmacotherapy<br>Behavioural support<br>Incentives for quitting<br>Distribution of resources	RCT	Intervention: *n* = 86<br>Control Group: yes	Knowledge<br>Consumption	Moderate
Schinke, Tepavac & Cole, 2000 [47]	N/A	USA	Native American	3 years	Education<br>Distribution of resources<br>Traditional rituals	RCT	Intervention: *n* = 1,396 (baseline)<br>*n* = 1,374 (6 mth follow-up)<br>*n* = 1,329 (18 mth)<br>*n* = 1,268 (30 mth)<br>*n* = 197 (3.5 years)<br>Control Group: yes	Initiation	Moderate
Sonoma County Indian Health Project, 2014[102]	Sonoma County Indian Health Project (SCIHP)	USA	American Indian/ Alaskan Native (AIAN)	6 years +	Brief intervention<br>Pharmacotherapy<br>Training health-care professionals Behavioural support<br>Quit courses<br>Distribution of resources<br>Smoking ban	Cross-sectional	*n* = 636	Quit rates	Weak

Study	Project Name	Location	Ethnicity	Intervention Length	Main Activities	Study Design	Sample Size	Outcomes Measured	Study Quality
Thomas, Johnston & Fitz, 2010 [21]	The Tobacco Project	Australia	Indigenous	15 months	Brief intervention<br>Pharmacotherapy<br>Media campaigns<br>Education<br>Distribution of resources<br>Traditional rituals<br>Sales restrictions<br>Smoking ban	Before and after	*n* = 23 (baseline), 13 (follow-up)	Community interest<br>Consumption	Moderate
Thomas et al., 2013 [48]	N/A	Australia	Aboriginal	7 months	Brief intervention<br>Pharmacotherapy<br>Training health-care professionals	Cross-sectional	*n* = 54	Consumption	Moderate
Walker et al., 2015 [43]	N/A	Australia and New Zealand	Māori or Australian Aboriginal/ Torres Strait Islander	3 months	Brief intervention<br>Pharmacotherapy<br>Behavioural support<br>Quitline	RCT	Intervention: *n* = 161<br>Control: *n* = 160	Smoke-free environments<br>Prevalence	Strong
Watson et al., 2011 [44]	New Zealand's Smoke-free Environments Amendment Act 2003 (SFEAA)	New Zealand	Maori	4 years	Smoking ban	Cross-sectional	*n* = 1252	Consumption<br>Smoke-free environments<br>Quit rates	Moderate
Weaver, 1999 [103]	N/A	USA	Native American	4-6 months	Distribution of resources<br>Traditional rituals	Before and After	*n* = 75 (baseline and follow-up)	Consumption	Weak
Weaver & Jackson, 2010 [45]	Healthy Living in Two Worlds	USA	Native American	5 weeks	Education<br>Traditional rituals	Before and After	*n* = 16 (baseline), 11 (follow-up)	Initiation<br>Consumption	Strong

Study	Project Name	Location	Ethnicity	Intervention Length	Main Activities	Study Design	Sample Size	Outcomes Measured	Study Quality
Wesche, Ryan & Carry, 2011 [62]	Peterborough County-City Health's "Choose to be SmokeFree" Tobacco Cessation Program	Canada	First Nations	Not specified	Peer support<br>Distribution of resources	Cross-sectional	Not reported	Smoking Abstinence	Weak
Wesche, Ryan & Carry, 2011 [62]	"Changing the Culture of Smoking" – A Community-Based Participatory Research (CBPR) Project, Inuvialuit Region	Canada	Inuit	5 years	Education<br>Distribution of resources	CBR	Not reported	Consumption<br>Smoke-free environments<br>Quit rates	Weak
Wesche, Ryan & Carry, 2011 [62]	"Stay Quit to Win Challenge" – Tobacco Control, Nunavik	Canada	Inuit	6 weeks	Education<br>Reinforcement of laws<br>Smoking bans	Before and After	*n* = 1273 (baseline and follow-up)	Smoke-free environments<br>Quit rates	Weak
Wesche, Ryan & Carry, 2011 [62]	Métis Nation British Columbia's Aboriginal ActNow BC Program	Canada	Métis	2 year	Behavioural support<br>Distribution of resources<br>Traditional rituals	Before and After	*n* = 72 leaders, 3100 participants (baseline)<br>*n* = 46 leaders, 140 participants (follow-up)	Consumption<br>Quit rates	Moderate

Study	Project Name	Location	Ethnicity	Intervention Length	Main Activities	Study Design	Sample Size	Outcomes Measured	Study Quality
Wilson et al., 2005 [104]	N/A	New Zealand	Maori	2 year	Behavioural support	Cohort Study	Not reported	Readiness to quit	Weak
Witmer et al., 2004 [59]	Traditions of the Heart	USA	Alaska Native and American Indian women	12 weeks	Behavioural support<br>Education	RCT	Intervention: *n* = 44 (baseline)<br>*n* = 27 (12 mth follow-up)<br>Control Group: yes	Consumption<br>Quit rates	Moderate

Intervention Characteristics (Descriptive Studies)

Study	Project Name	Location	Ethnicity	Intervention Length	Main Activities
Choi et al., 2011 [105]	All Nations Breath of Life	USA	American Indian	2 year	Incentives for quitting<br>Peer support<br>Events
Horn et al., 2008 [79]	American Indian Not on Tobacco (N-O-T) program	USA	American Indian	Not specified	Behavioural support<br>Distribution of resources
Horn et al., 2009 [106]	American Indian Not on Tobacco (N-O-T) program	USA	American Indian	1 year	Not specified
Johnston et al., 2010 [107]	N/A	Australia and New Zealand	Indigenous peoples in Australia and New Zealand	3 months	Brief intervention<br>Pharmacotherapy<br>Training health-care professionals Behavioural support<br>Distribution of resources

Study	Project Name	Location	Ethnicity	Intervention Length	Main Activities
Makosky et al., 2008 [108]	All Nations Breath Of Life	USA	American Indians\Alaska Natives		Pharmacotherapy<br>Behavioural support<br>Distribution of resources
Marley et al., 2014 [109]	Be Our Ally Beat Smoking (BOABS) study	Australia	Aboriginal peoples and Torres Strait Islanders	1 year	Brief intervention<br>Pharmacotherapy
Robertson, 2010 [26]	Top-End Tobacco Project	Australia	Indigenous	Not specified	Brief intervention<br>Pharmacotherapy<br>Education<br>Quit support for health professionals Smoking ban
Stefanich et al., 2005 [110]	Traditions of the Heart	USA	Alaska Native	3 months	Behavioural support<br>Education<br>Distribution of resources

Note: RCT = randomized controlled trial
Non RCT = non-randomized controlled trial
CBR = community based research
Mth = month
