# Supplementary material for: Effective strategies to reduce commercial tobacco use in Indigenous communities globally: A systematic review
Source: BMC Public Health. 2016 Jan 11;16:21. doi: 10.1186/s12889-015-2645-x (PMC4710008; doi:10.1186/s12889-015-2645-x)
Supplement: Supplementary file 3 — Quality Assessment Tool. (PDF 71 kb) [file 12889_2015_2645_MOESM3_ESM.pdf]

Additional File 3: Quality Assessment Tool

1.	**Rigour of the evaluation methods including design, implementation and analysis**
1.1	Is the study design evident and appropriate to research questions?<br>**Yes,** design and methodology are clearly described and appropriate to research questions<br>**Partial,** study design is evident but does not fully reflect research questions<br>**No,** design and methodology are not described OR inappropriate for research questions
1.2	Sampling<br>Quantitative:<br>**Yes,** sample size is both described and justified<br>**Partial,** sample size is described but not justified<br>**No,** sample size is not described nor justified<br><br>Qualitative:<br>**Yes,** the sample size is described AND participants are selected using purposeful sampling strategies<br>**Partial,** the sample size is described OR participants are selected using purposeful sampling strategies<br>**No,** sample size is not described AND/OR the authors do not use purposeful sampling strategies
1.3	Are the paper's conclusions supported by the results? Is it clear that the data justify the conclusions drawn?<br>**Yes,** conclusions are well supported by the data (reported outcomes), any speculation is explicit<br>**Partial,** major conclusions are supported, but speculation is not flagged as much<br>**No,** conclusions are not supported by the data (ex. conclusions are made on the basis on non-significant results without explicit attention) OR are missing
1.4	Are the methods/outcome measures used reliable for the research question and context?<br>Quantitative: Do the results provide consistent results?<br>**Yes,** instruments have good reliability AND authors describe instruments that have established reliability (internal consistency or test-retest)<br>**Partial,** good face reliability but authors do not explicitly express established reliability<br>**No,** reliability is unreported, unreferenced and instruments lack face reliability<br><br>Qualitative:<br>**Yes,** accurate and adequate documentation of changes in the setting/ phenomena being studied were described AND researcher locates themselves with respect to the phenomena being studied<br>**Partial,** accurate and adequate documentation of change in the setting/phenomena being studied were described OR researcher locates themselves with respect to the phenomena being studied<br>**No,** there is no documentation of changes and the research does not locate themselves with respect to the phenomena being studied
2.	**Strength of the evidence**
2.1	Is the response rate reasonable?<br>Quantitative:<br>**Yes,** sample size allows for adequate statistical power<br>**Partial,** sample size is not ideal but adequate<br>**No,** sample size is inadequate leading to statistically significant or low power responses<br><br>Qualitative:<br>**Yes,** the sample is ideally representative of the diversity and breadth of the sample population AND sample size reached saturation<br>**Partial,** the sample is not ideal but an adequate representation of the diversity and breadth of the sample population AND/OR sample size did not reach saturation<br>**No,** the sample is not representative of the population AND sample size did not reach saturation

2.2	Are threats to internal validity (the extent to which the program leads to the measured changes) assessed and accounted for where possible?<br>Quantitative:<br>**Yes,** the authors describe the established validity of the instrument OR authors both address and account for threats to validity (ex. Confounding factors) in their design<br>**Partial,** authors are not explicit in threats to internal validity although good face validity is evident OR address factors but do not account for them in study design.<br>**No,** Validity is unreported, unreferenced and instruments lack face validity. Very uncertain if the program has led to the measured changes (No cause and effect).<br><br>Qualitative: Are results credible? Is more than one method used to support results (Triangulation)<br>**Yes,** the results of qualitative research are credible or believable from the perspective of the participant in the research. (The researchers checked with other researchers or participants to ensure themes are accurate) AND more than one appropriate method was used to support results OR peer debriefing/ external auditing of research protocol/ results occurred<br>**Partial,** there is some evidence that the results are credible or believable from the perspective of the participant in the research OR one method was used, but only one was appropriate OR peer debriefing/ external auditing of research protocol is not evident<br>**No,** there is no evidence that the results are credible or believable from the perspective of the participant in the research AND no triangulation occurred or peer debriefing/ external auditing is missing
2.3	External Validity (Answer depending on whether research was quantitative vs. qualitative):<br>Quantitative:<br>**Yes,** the authors take into account threats to external validity into the research design. This may mean that authors account for participant differences by matching control and intervention groups/ baseline and follow up groups and keep dropout levels low OR randomization<br>**Partial,** authors recognize that study cannot be generalized but do not work to improve study design and decrease threats to external validity<br>**No,** results are not generalizable and author does not recognize the threats to external validity/ generalizability<br><br>Qualitative:<br>**Yes,** authors provided rich, thick, detailed descriptions so that anyone interested in transferability will have a solid framework for comparison AND assumptions that were central to the research are described<br>**Partial,** authors provided an adequate description OR assumptions that were central to the research are described<br>**No,** authors provide no detailed description of methods or results for comparison AND assumptions that were central to the research are missing
2.4	Is there reflexivity of the account? Are limitations, biases, and perspectives/identity declared?<br>**Yes,** critical discussion is included and makes references to limitations, biases, perspectives/identity and conflicts of interest<br>**Partial,** critical discussion is included for one or two but does not fully address all of the above<br>**No,** no critical discussion is included
3.	**Relevance to community**
3.1	Is the study design/ measures in keeping with local community values/beliefs/knowledge systems?<br>**Yes,** evidence provided explicitly in the text (look for: where did evaluation take place, who collected evaluation data?)<br>**Partial,** hints of it in text therefore assumption made by reviewers that evidence is present<br>**No,** nothing was said or it was said that it was not done

3.2 Is the study design/ measures in keeping with local priorities/aims (e.g. community articulated need for this evaluation)?<br>**Yes,** evidence provided explicitly in text (look for: stakeholder involvement, hiring local Aboriginal research staff, capacity building)<br>**Partial,** hints of it in text therefore assumption made by reviewers that evidence is present<br>**No,** nothing was said or it was said that it was not done
3.3 Is the underlying theory (of evaluation) relevant to the community?<br>**Yes,** evidence provided explicitly in text (evidence of themes of reciprocity, two-eyed seeing, recognizing capacity building)<br>**Partial,** hints of it in text therefore assumption made by reviewers that evidence is present<br>**No,** nothing was said or it was said that it was not done
3.4 Is the study protocol (data collection method) vetted by local community members?<br>**Yes,** evidence provided explicitly in text (look for advisory council guidance and review of tool,<br>**Partial,** hints of it in text therefore assumption made by reviewers that evidence is present<br>**No,** nothing was said or it was said that it was not done
